# Mechanisms of Anesthetic Action and Neurotoxicity: Lessons from Molluscs

**DOI:** 10.3389/fphys.2017.01138

**Published:** 2018-01-23

**Authors:** Ryden Armstrong, Saba Riaz, Sean Hasan, Fahad Iqbal, Tiffany Rice, Naweed Syed

**Affiliations:** ^1^Vi Riddell Pain Program, Alberta Children's Hospital Research Institute, Hotchkiss Brain Institute, Cumming School of Medicine, University of Calgary, Calgary, AB, Canada; ^2^Department of Anesthesia, Alberta Children's Hospital, University of Calgary, Calgary, AB, Canada

**Keywords:** anesthesia, neurons, synapses, *Lymnaea*, molluscs, synaptic transmission, cytotoxicity

## Abstract

Anesthesia is a prerequisite for most surgical procedures in both animals and humans. Significant strides have been made in search of effective and safer compounds that elicit rapid induction and recovery from anesthesia. However, recent studies have highlighted possible negative effects of several anesthetic agents on the developing brain. The precise nature of this cytotoxicity remains to be determined mainly due to the complexity and the intricacies of the mammalian brain. Various invertebrates have contributed significantly toward our understanding of how both local and general anesthetics affect intrinsic membrane and synaptic properties. Moreover, the ability to reconstruct *in vitro* synapses between individually identifiable pre- and postsynaptic neurons is a unique characteristic of molluscan neurons allowing us to ask fundamental questions vis-à-vis the long-term effects of anesthetics on neuronal viability and synaptic connectivity. Here, we highlight some of the salient aspects of various molluscan organisms and their contributions toward our understanding of the fundamental mechanisms underlying the actions of anesthetic agents as well as their potential detrimental effects on neuronal growth and synaptic connectivity. We also present some novel preliminary data regarding a newer anesthetic agent, dexmedetomidine, and its effects on synaptic transmission between *Lymnaea* neurons. The findings presented here underscore the importance of invertebrates for research in the field of anesthesiology while highlighting their relevance to both vertebrates and humans.

## Introduction

A wide variety of anesthetic compounds are safely administered to patients of all ages every year. General anesthetic agents can be broadly classified as either volatile inhaled compounds, or intravenously administered compounds. Structurally, there is a wide range of volatile anesthetic compounds, from simple diatomic compounds such as nitrous oxide, to fluorinated ethers including the modern volatile anesthetics isoflurane, sevoflurane, and desflurane. Intravenous anesthetics are even more varied, from simple hydrocarbons to steroid compounds (Harrison et al., 1987). Common intravenous anesthetics include propofol and ketamine. Since there is a broad spectrum of compounds exhibiting anesthetic effects, there remains significant uncertainty as to how and where these compounds act at the molecular and cellular levels.

Although anesthetic agents are typically regarded as safe to administer to patients with proper monitoring, in recent years, evidence has emerged regarding potential anesthestic-related neurotoxicity. Specifically, studies have shown that general anesthetics may impair nervous system development in animals exposed to these agents during periods of peak neurodevelopment and this has raised concern for young children (Ikonomidou et al., 1999; Flick et al., 2011; Armstrong, 2016; Vutskits and Davidson, 2017; Walters and Paule, 2017). As such, there remains the possibility that general anesthetic exposure in children may lead to long-term cognitive impairment and learning and memory deficits. However, a clear consensus on the precise impact that general anesthetics have on the developing human nervous system and on the kind of protective strategies that may be used to mitigate this risk is lacking. In addition, despite their widespread use, the specific mechanisms of action of general anesthetics also remain unclear (Armstrong, 2016). Molluscs have proven invaluable for teasing out some of the direct effects of general anesthetics, which we report here.

This current review provides a brief overview of molluscan studies that have helped to shape our understanding of both the actions of anesthetic agents as well as their possible detrimental effects on neurons. Broadly speaking, these mollusc studies were the first fundamental investigations into anesthetic mechanisms of action that predated and laid the groundwork for future mammalian studies. We highlight the important roles that molluscs continue to play in the examination of anesthetic-induced neurotoxicity, currently a very active research area in both vertebrates and the clinical setting. In addition, we systematically evaluate the subcellular impact of modern general anesthetics on neuronal excitability, viability, and connectivity between developing neurons. While many of the findings reviewed here emanate from research conducted on various molluscs, particularly the fresh water snail *Lymnaea*, these data have nevertheless stood the test of time as it pertains to vertebrates (Xu et al., 2016).

## Molluscs are valuable tools for fundamental research on general anesthetics

Notwithstanding considerable efforts, the underlying mechanisms of anesthetic actions, and their potential long-term side effects remain largely unknown. These limitations are due, in part, to the complexity of mammalian models, which are comprised of complex, highly interconnected neuronal networks. Molluscs, on the other hand, are well-suited for researching the fundamental mechanisms of action and neurodegenerative effects of anesthetic agents due to their relatively simple neuronal networks mediating well-defined, simple, behaviors. Several molluscs have been used, including the squid *Loligo forbesi* and the pond snail *Lymnaea stagnalis*.

Some of the earliest work to understand the fundamentals of neuronal function was performed in the squid giant axon. Similarly, early work to understand the mechanisms of action of anesthetic agents also used this easy to manipulate system. Shrivastav et al. performed some of the initial work on *L. forbesi* (Shrivastav et al., 1976). They exposed a giant squid axon to the volatile anesthetic halothane and recorded membrane depolarization at low anesthetic concentrations. They observed a similar depolarizing effect with the volatile anesthetic trichloroethylene, which also increased the threshold potential for action potential firing, and reduced the amplitude of resulting action potentials (Shrivastav et al., 1976). This resulted in the build-up of sodium ions within the cell and an inhibition of potassium permeability across the membrane, creating an intracellular environment that permitted only action potentials with suppressed peak amplitude. These early studies suggested that general anesthetics may transiently block action potential conduction in axons. After the removal of the anesthetic, the action potentials returned to control amplitude, highlighting the transient nature of general anesthetic effect (Shrivastav et al., 1976). Shrivastav later tested the effects of the intravenous anesthetic drug ketamine on squid giant axons (Shrivastav, 1977). Exogenous application of the drug suppressed the peak ion conductance during action potentials similar to that of volatile anesthetics, further validating a hypothesis of anesthetic-induced sodium ion build-up inside the cell (Shrivastav, 1977).

Several years later in 1983, a study by Haydon and Urban expanded on Shrivastav group's work by testing the effects of a range of volatile anesthetics (methoxyflurane, halothane, dichloromethane, and chloroform) on the sodium currents of squid giant axons (Haydon and Urban, 1983). Exposure to the anesthetic agents depolarized the neurons and reduced peak sodium ion conductance, impairing action potential generation and propagation. Interestingly, higher lipid solubility of anesthetic agents correlated with a more rapid onset of effect, suggesting that the lipid solubility of a compound may play an important role in its anesthetic action (Haydon and Urban, 1983). This correlated well with very early observations summarized in the Meyer-Overton hypothesis, which states that the potency of an anesthetic agent is directly related to its lipid solubility (Meyer, 1899; Overton, 1901). Expanding on this work, Haydon and Urban tested many other anesthetic agents, including non-polar molecules, alkanols of various lengths, and a range of volatile anesthetics, on squid giant axons and measured ion currents. They observed that all anesthetics diminished both the potassium and sodium currents of the axons significantly in a manner that correlated broadly with the agent's lipid solubility (Haydon and Urban, 1986). Furthermore, this finding supported Shrivastav et al.'s earlier suggestion that potassium permeability was suppressed after exposure to general anesthetics and generalized it to a wider range of compounds.

Furthering previous work on ion currents under the influence of anesthetics, Haydon and Simon published a study in 1988 where they measured the threshold potential of a squid giant axon under the influence of clinically relevant concentrations of many general anesthetics (Haydon and Simon, 1988). The action potential threshold was changed variably: certain agents reduced the threshold, while others increased it. General anesthetic agents all reversibly depolarized the resting membrane potential slightly by roughly 1–5 mV. The actions of all agents were fully reversible, although some subsided immediately whereas others required extensive washout (Haydon and Simon, 1988).

There were several key parallels between the experiments of Shirvastav et al., Haydon and Urban, and the study performed by Haydon and Simon in *L. forbesi*. Specifically, all of these studies demonstrated that the giant squid axons were slightly depolarized due to general anesthetic exposure. Shirvastav et al. hypothesized that this was achieved due to the accumulation of sodium ions inside the tested neurons concurrent with the inhibition of potassium ion movement (Shrivastav et al., 1976). This meant that the cell remained depolarized and unable to repolarize completely until the complete removal of the anesthetic agent from the bathing solution. Haydon and Urban then confirmed this observation by measuring these sodium and potassium currents directly (Haydon and Urban, 1983, 1986).

Another mollusc, the fresh water pond snail *L. stagnalis*, rose to prominence in the early to mid 1980s when Winlow's laboratory conducted a series of behavioral studies on the effects of inhalational anesthetics on freely behaving animals. Specifically, Girdlestone et al. demonstrated that halothane and isoflurane, when used in clinically relevant concentrations, brought about a state of anesthesia in freely behaving *Lymnaea* (McCrohan et al., 1987; Girdlestone et al., 1989). Similarly, Franks and Lieb reinforced *Lymnaea*'s effectiveness in anesthesia research in 1990, when they used this invertebrate to demonstrate that general anesthetics act on proteins rather than on the lipid bilayer as their primary target (Franks and Lieb, 1991). Previous research by Harris and Groh had shown that a temperature change of merely 1°C caused a change in membrane potential across the lipid bilayer roughly equal to that caused by general anesthetics, thus challenging the notion that general anesthetic agents solely act on the lipid bilayer as their potential target site (Harris and Groh, 1985). Correspondingly, Franks and Lieb noted that different general anesthetics competitively inhibited an anesthetic-sensitive protein, Luciferase, at the same ED_50_ concentrations required to put a patient into the state of general anesthesia (Franks and Lieb, 1990). This result strongly suggested that the most likely primary sites of action for general anesthetics are protein molecules rather than the lipid bilayer (Franks and Lieb, 1981).

Franks and Lieb further demonstrated the cellular specificity of general anesthetics by exposing several *Lymnaea* neurons to halothane, and observing that only a single cell had its electrophysiological properties altered after exposure, while the other cells remained unaffected (Franks and Lieb, 1988). Interestingly, Franks and Lieb reported that the affected cell exhibited an anesthetic-activated potassium channel, which the neighboring anesthetic-insensitive cells did not possess (Franks and Lieb, 1988, 1990). Activation of this channel resulted in hyperpolarization of the cell. These results stand in contrast to those observed in the squid, whereby potassium conductance was reduced after anesthetic exposure (Shrivastav et al., 1976). This discrepancy could perhaps be attributed to cell specific differences in *Lymnaea* channel proteins, which may be absent in the squid.

Franks and Lieb then went on to determine where exactly on the neuron the halothane-activated potassium conductance [I_K(An)_] was activated (Franks and Lieb, 1991). They suggested that since halothane reversibly inhibited the action potentials (and by extension synaptic transmission in the isolated cell), then the halothane sensitivity may likely be greatest at an area rich with synaptic connections (Franks and Lieb, 1991). Thus, synaptic stimulation could possibly modulate the I_K(An)_ conductance *in vivo*. Concurrently, the neuropeptide FMRFamide, which acts as a neuromodulator in molluscs, inhibited the I_K(An)_ conductance in isolated neurons, suggesting a certain measure of control over potassium flow (Franks and Lieb, 1991). Neuropeptides such as FMRFamide act both presynaptically and postsynaptically, and modulate communication between neurons by altering acetylcholine release or response. Later in 2007, Andres-Enguix et al. advanced these observations regarding the halothane-activated potassium conductance made by Franks and Lieb (Franks and Lieb, 1988, 1990, 1991; Andres-Enguix et al., 2007). Andres-Enguix et al. cloned the two-pore domain potassium (K+) channel, which exhibited significant similarities to the anesthetic-activated potassium channel identified in *Lymnaea* and was found to have an amino acid necessary for anesthetic binding (Franks and Lieb, 1988, 1991; Andres-Enguix et al., 2007). This suggested that the identified two-pore domain potassium channel may be a potential target for general inhalational anesthetics (Andres-Enguix et al., 2007). Furthermore, these results strongly suggested that there are specific moieties on proteins that are responsible for directly binding anesthetics and modulating their actions.

Focusing on synapses, which play fundamental roles in neuronal function, learning, and neuroplasticity, McKenzie et al. documented the effects of several different general anesthetics on *Lymnaea* nicotinic acetylcholine receptors (nAChR), which are the postsynaptic receptors for acetylcholine (ACh). All of the anesthetic agents suppressed the cells' ion currents through the nAChR, further suggesting that anesthetics target proteins. In support of this, they also mapped potential general anesthetic binding sites on the neuronal nicotinic AChR (McKenzie et al., 1995). These correlations strongly suggested that neuronal nicotinic AChRs might play a role in the molecular mechanism of general anesthetics—with specific impact on neuronal networks involved in learning and memory.

In 1996, Spencer et al. shifted the focus of general anesthesia research by using *L. stagnalis* to test the effects of halothane on inhibitory and excitatory peptidergic synapses. Because it is difficult, if not impossible, even in relatively simple molluscs, to decipher the direct vs. indirect effects of anesthetic at the level of single or synaptic paired cells *in vivo*, Spencer et al. employed the unique ability of the *Lymnaea* nervous system to reconstitute functional synaptic networks *in vitro*. As a result, they were able to compare and contrast the effects of clinically relevant concentrations of halothane on reconstructed synaptic networks (Spencer et al., 1995). Halothane suppressed excitatory synapses at lower concentrations than those that were required to suppress inhibitory synaptic transmission, in effect causing a net increase in inhibitory synaptic transmission at lower concentrations of halothane (Spencer et al., 1996). This study was important because it showed that halothane likely affected not only presynaptic, but also postsynaptic responses (Spencer et al., 1996). Most significantly, this study demonstrated for the first time that general anesthetic agents may also exert their effects on peptidergic modulated synapses—in addition to classical neurotransmitter synapses. These studies thus broadened the scope of anesthetic actions beyond classical transmitters to include peptidergic communication in the brain.

With the emergence of sevoflurane as a modern volatile anesthetic agent, Hamakawa et al. provided the first direct evidence regarding the fast acting actions of this inhalational agent on the inhibition of synaptic transmission between *in vitro* reconstructed *Lymnaea* synapses followed by a rapid recovery. This study also demonstrated that like halothane, sevoflurane likely suppresses synaptic transmission between identified *Lymnaea* neurons at both presynaptic and postsynaptic sites (Hamakawa et al., 1999).

Intravenous anesthetic agents have also been studied in *Lymnaea*. Woodall and McCrohan compared the effects of the intravenous anesthetics propofol and ketamine on the neuronal and behavioral activity of *L. stagnalis* (Woodall and McCrohan, 2000). Unlike inhalational anesthetic agents (Franks and Lieb, 1988), these intravenous compounds did not induce anesthesia at the tested concentrations in freely moving *Lymnaea* nor did they affect the resting membrane potential of neurons (Woodall and McCrohan, 2000). Rather, both agents suppressed the amplitude of the first action potential occurring after hyperpolarization of only a single specific identified neuron, which acts as part of the respiratory pattern generator in *Lymnaea* (Syed et al., 1990). Interestingly, in other identified neurons, elicited burst spike activity was increased by propofol, which suggested a possible transient excitatory effect on the neurons and by extension, a possible explanation for the snail's hyperactivity observed in the study (Woodall and McCrohan, 2000). Since these compounds are highly effective at inducing anesthesia in humans, this finding highlights that these specific agents may target different neuronal structures than the volatile agents.

In addition to using *Lymnaea* as a system for studying the mechanism of action of anesthetic agents, these molluscs have been used to help elucidate possible detrimental effects of exposure to anesthetic medications. Furthering their initial work, Woodall et al. asked whether the chronic application of the intravenous general anesthetic agent propofol would be detrimental to the growth of injured neurons and if it would also block synaptogenesis between *Lymnaea* neurons. They noted that only the postsynaptic acetylcholine response was inhibited as a result of propofol exposure (Woodall et al., 2003). Taken together with their previous work (Woodall and McCrohan, 2000), this indicated that propofol may not have an impact on a cell's intrinsic electrophysiological properties but rather affects its ability to communicate with other neurons. Furthermore, propofol did not inhibit neurite outgrowth; however, long-term exposure reversibly blocked the initial formation of synapses, perhaps by blunting the postsynaptic acetylcholine responses (Woodall et al., 2003). Similarly, Naruo et al. studied the effects of a commonly used volatile anesthetic, sevoflurane, on a well-characterized *Lymnaea stagnalis* synapse pairing [visceral dorsal 4 (VD4)–left pedal dorsal 1 (LPeD1)] (Naruo et al., 2005). They observed that the agent's effects on synaptic transmission involved a blunting of the postsynaptic nicotinic acetylcholine receptor response, similar to the findings with propofol by Woodall et al. This study also asked whether volatile anesthetics affect short-term synaptic plasticity. Specifically, by pairing neurons they were able to consistently test the synapse's post-tetanic potentiation, which is thought to form the basis of working memory (Luk et al., 2011). Naruo's study was the first to demonstrate that whereas sevoflurane blocked synaptic transmission in clinically relevant concentrations, it did not affect post-tetanic potentiation (Naruo et al., 2005).

Later, Onizuka et al. exposed *Lymnaea* neurons to commonly used general and local anesthetics to observe their effects on synaptogenesis, neurite growth and synaptic transmission (Onizuka et al., 2005b). Similar to previous studies (Woodall et al., 2003), they showed that general anesthetic agents depressed excitatory cholinergic synaptic transmission postsynaptically, but did not inhibit neurite outgrowth or synaptogenesis. Conversely, the local anesthetic agents depressed excitatory cholinergic synaptic transmission presynaptically, and inhibited both neurite outgrowth and synaptogenesis (Onizuka et al., 2005b). However, the exact reason as to why the local anesthetic agents exhibited such dramatic effects is yet to be attributed to any specific inherent neurotoxicity, or to the inhibition of presynaptic mechanisms. Finally, a 2008 study by Browning and Lukowiak shed light on further possible detrimental effects that general anesthetics might have on memory formation. In this study, they determined that ketamine only inhibited long-term memory formation, and not its shorter-term counterparts (Browning and Lukowiak, 2008), which they attributed to be due to ketamine's interference with mRNA transcription required for the formation and storage of memories. This study highlights the potential for further studies that can be conducted to examine transcriptome changes after exposure to anesthetic compounds.

**Figure 1:**
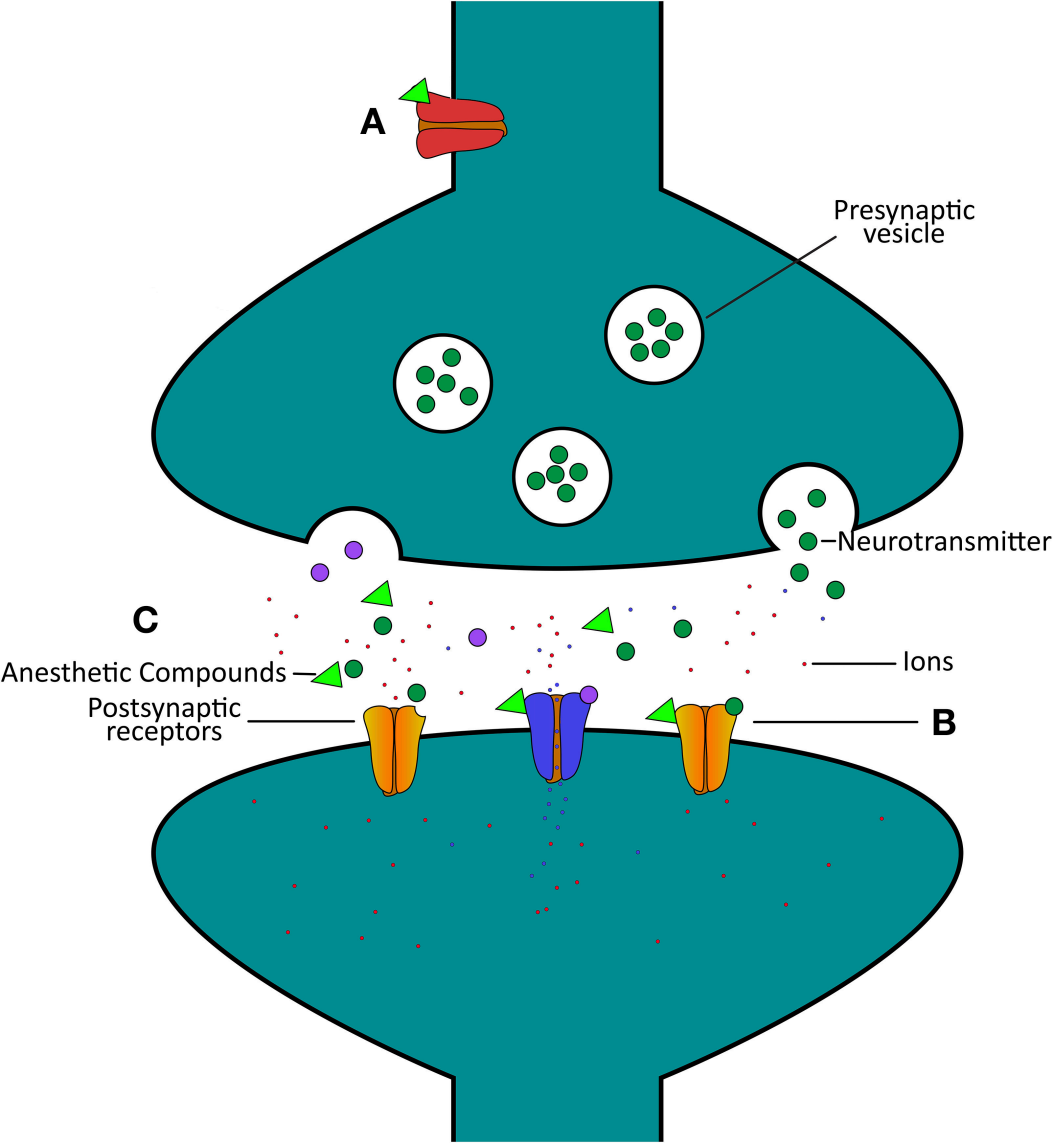
Simplified schematic highlighting certain actions of general anesthetics on the *Lymnaea* synapse. **(A)** The anesthetic (green triangle)-activated potassium channel [red channel, I_K(An)_] hyperpolarizes neurons, and thus will cause a reduction in presynaptic neurotransmitter release (Franks and Lieb, 1991). **(B)** A wide range of general anesthetics have been found to inhibit the response of the acetylcholine receptor to neurotransmitter, thus inhibiting synaptic communication (McKenzie et al., 1995). **(C)** Represents various potential target sites of anesthetic actions on neurons and their synaptic communication, from presynaptic release of neurotransmitter to postsynaptic response. Excitatory postsynaptic receptors for acetylcholine (yellow, excitatory, closed channels) are closed by anesthetic (green triangle) exposure, but at similar concentrations inhibitory postsynaptic channels (blue, inhibitory, open channels) remain open. As such, synaptic communication is preferentially suppressed in excitatory synapses as compared to inhibitory synapses at similar anesthetic concentrations (Spencer et al., 1996).

Taken together, these molluscan studies provide direct evidence that in the absence of network complexity and glia cells, anesthetics cause a net reduction in excitatory neurotransmission between neurons. Early work in *Loligo* showed that general anesthetics transiently block action potential amplitude and propagation down axons, likely by trapping sodium ions within cells. Further work in *Lymnaea* fleshed out these findings. Specifically, work showed that volatile general anesthetic compounds directly interact with ion channel proteins, in particular a potassium channel that hyperpolarized neurons, preventing neurotransmitter release. Subsequent work then showed that general anesthetics also directly target and suppress ion movement through the excitatory acetylcholine receptor. These studies (Franks and Lieb, 1991; McKenzie et al., 1995) thus show that some general anesthetics target both presynaptic and postsynaptic sites on neurons. These studies also highlight the potential target sites of anesthetic actions that include classical and peptidergic neurotransmitter synapses. Finally, the data from various studies on *Lymnaea* demonstrate that chronic exposure of cultured neurons to anesthetic compounds might render neuronal growth and synaptic connectivity dysfunctional. This work laid valuable foundations for potential molecular targets of anesthetics. Indeed, anesthetic agents are now believed to target many more channels than merely the nAChR, including gamma-aminobutyric acid (GABA)_A_, *N*-methyl-D-aspartate (NMDA), α-amino-3-hydroxy-5-methyl-4-isoxazolepropionic acid (AMPA), and serotonin receptors, in a manner that preferentially enhances inhibitory transmission (Harris et al., 1995; Campagna et al., 2003; Hemmings et al., 2005). Figure 1 shows a simplified diagram of where general anesthetics may act on the *Lymnaea* synapse.

## Molluscs are valuable tools for local anesthesia research

Similar to general anesthetics, local anesthetic agents have been extensively tested in molluscs to help elucidate their specific mechanisms of action. Interestingly, recent studies have also shown that these anesthetic agents may have detrimental side effects on neuron function in invertebrates.

The first of such studies to elucidate the mechanisms of action of local anesthetics in invertebrates was in 1978 when Yeh exposed a *Loligo pealei* axon to various sodium channel blocking local anesthetics (Yeh, 1978). Yeh showed that the anesthetics produced a blockade of the sodium channel that was dependent on both the membrane potential and the frequency of any action potentials currently passing through the axon using the sodium channels, thus explaining how local anesthetics may perturb transmission of the action potential through nerve fibers. Later, in 1981, Ohki et al. showed that the relative solubility of local anesthetics in the lipid bilayer of axon membranes played an important role in their action (Ohki et al., 1981). This study was further confirmed by Wang et al. who showed that the hydrophobicity of local anesthetics was needed to interact with their binding domains on the sodium channel (Wang et al., 1982). Furthermore, they noted that the greater the hydrophobic characteristic of the local anesthetic, the greater the blockade of the sodium channel. Interestingly, this observation matches that seen with general anesthetics, according to the Meyer-Overton hypothesis.

Starmer et al. subsequently tested whether local anesthetic-mediated sodium channel blockade was the result of local anesthetic compounds being trapped within a cell and then binding receptors from within (Starmer et al., 1986). In the giant squid axon, they observed that local anesthetics bound to the interior of the ion channel, with binding being dependent on the membrane potential. As such, the degree of sodium ion blockade varied with the membrane voltages.

Other molluscs, such as *L. stagnalis*, have allowed further insights into local anesthetic mechanisms of action and possible toxic side effects on neurons. In 2003, Kasaba et al. showed impairments in neuronal growth cones and changes in neurite morphology as a result of local anesthetic exposure. The group showed that mepivacaine and procaine resulted in the least harmful effects, whereas lidocaine was the most toxic (Kasaba et al., 2003). Further studies also noted a significant increase in intracellular calcium (Ca^2+^) concentration after local anesthetic exposure (Kasaba et al., 2006; Kasaba, 2007). Interestingly, the morphological damage and calcium concentration were not well-correlated suggesting that there was yet another mechanism of action underlying the compromised neuronal growth (Kasaba et al., 2006). To further test this link between calcium and neurite growth, Kasaba et al. exposed *Lymnaea* neurons to lidocaine in the presence or absence of a calcium chelator. They found no morphological damage despite increased intracellullar Ca^2+^ concentration at low lidocaine concentrations. However, as the lidocaine concentration increased so too did the calcium concentration, causing an increase in the observed morphological damage. When combined with a calcium chelator, the resulting damage was not reduced, indicating that the damaging effects were likely not mediated solely through calcium ion accumulation (Kasaba, 2007).

In 2007, Kasaba et al. focused on another aspect of lidocaine exposure in *Lymnaea* by examining the burst action potential spikes of neurons. As the drug's concentration increased, the bursts of action potential spikes got weaker indicating an inhibition in synaptic transmission (Kasaba et al., 2007). Further studies have been used to dissect this phenomenon. Onizuka's group demonstrated that exposing a *L. stagnalis* neuron to lidocaine resulted in a transient increase in intracellular sodium concentration, which might explain the initial excitatory phase observed following the anesthetic exposure (Onizuka et al., 2004). With the addition of the potent sodium channel blocker tetrodotoxin (TTX), there was no change in intracellular sodium concentrations, indicating that the very sodium channels ultimately blocked by lidocaine may underlie the initial rise in sodium concentration. They subsequently noted that lidocaine had a dose-dependent effect (Onizuka et al., 2005a). Further fleshing out these findings, Onizuka et al. demonstrated that lidocaine decreased the excitatory postsynaptic potential response to exogenously applied acetylcholine (Onizuka et al., 2008).

In a study designed to investigate whether or not local anesthetics alter neuron morphology, Onizuka et al. reported the formation of bullae and blebs, as well as increased cell size—which indicates cells undergoing either apoptosis or necrosis—in neurons exposed to lidocaine in a dose-dependent manner (Onizuka et al., 2012b). This may be due to the irreversible depolarization of the resting membrane potential they noted as a result of the lidocaine exposure, which may cause irreversible damage to the neurons (Onizuka et al., 2012b). In addition, Onizuka et al. demonstrated that neuron exposure to lidocaine inhibited neurotrophic growth factor (NGF)-induced axon growth and synaptic excitation (Onizuka et al., 2012a).

In summary, molluscs have proven useful to determine the mechanism of action of local anesthetics. Molluscs have allowed us to precisely identify the intracellular portion of the voltage-gated sodium channel as the primary target of local anesthetics. These findings have been borne out in other studies (Butterworth and Strichartz, 1990; Scholz, 2002). Further work has demonstrated that local anesthetics may be neurotoxic. These more recent studies collectively provide evidence for possible detrimental effects of local anesthetics on snail neurons. Lidocaine has been shown to have substantial effects on neuronal development. Lidocaine may be directly neurotoxic by suppressing synaptic function or by blocking the sodium channels that allow action potential propagation to the synapse. Exposure to lidocaine and its derivatives appears to affect the cell to a point where it is unable to self-regulate its intrinsic membrane properties. The interference in sodium channel flux may thus cause growth abnormalities in both the neurite and growth cones.

## Clinical research related to anesthetic-induced neurotoxicity

Research conducted in various molluscs has established many of the mechanisms of action of anesthetic agents. In addition, molluscs provide the ability to study relatively simple neuronal networks and to reconstruct individual synapses, which may allow further understanding of the mechanisms of potential anesthetic neurotoxicity. Recent work has shown that there exists robust evidence from vertebrate animals that both intravenous and inhalational anesthetic agents may be neurotoxic and lead to deficits in behavior, cognition, learning, and memory (Walters and Paule, 2017). Results have been consistent across multiple species, using various drugs and dosing paradigms. Early studies in neonatal rats suggested that exposure to anesthetic agents that are NMDA receptor antagonists or GABA_A_ receptor agonists resulted in cellular apoptosis and neurodegeneration in young brains (Ikonomidou et al., 1999, 2000; Olney et al., 2000). Similar findings were described when postnatal day 7 rats were given a combination of midazolam, nitrous oxide and isoflurane for 6 h, causing neuronal degeneration, deficits in synaptic function and persistent learning and memory impairments (Jevtovic-Todorovic et al., 2003). Numerous subsequent studies have corroborated these findings. For example, young rodents exposed to volatile anesthetics including sevoflurane and isoflurane were found to have deficits in long-term memory (Ramage et al., 2013). Rat cortical neurons exposed to either sevoflurane or desflurane demonstrated increased cell death, decreased neurite outgrowth and compromised mitochondrial integrity and synaptic function (Xu et al., 2016). Studies involving neonatal rodent exposure to intravenous anesthetics such as ketamine and propofol have also described detrimental effects on neuronal survival, dendritic spine density, and memory (Cattano et al., 2008; Pesić et al., 2009; Briner et al., 2011; Huang et al., 2012; Yu et al., 2013).

Findings in non-human primates have strengthened the animal literature in the field. Rhesus monkeys exposed *in utero* or as young neonates to various intravenous or inhalational anesthetics showed increased neuronal and glial apoptosis (Brambrink et al., 2010, 2012; Creeley et al., 2013, 2014). Five to six day old rhesus monkeys exposed to 24 h of ketamine continued to demonstrate cognitive impairments until at least three and one half years of age (Paule et al., 2011). The period of greatest vulnerability to the neurotoxic effects of anesthetic agents appears to correlate with the developmental stage of synaptogenesis, which in humans is thought to occur during the third trimester of pregnancy and the first 2 years of life (Hansen, 2015; Jevtovic-Todorovic and Brambrick, 2017; Walters and Paule, 2017).

Given the preponderance of evidence supporting anesthetic neurotoxicity in the animal literature, concerns have arisen regarding the exposure of human pediatric patients to these agents. However, despite convincing findings in the animal literature, human studies to date have provided limited and inconsistent results with respect to the effects of anesthetic agents on the developing brain (Bartels et al., 2009; DiMaggio et al., 2009; Kalkman et al., 2009; Wilder et al., 2009; Flick et al., 2011; Ing et al., 2012; Davidson et al., 2016; O'Leary et al., 2016; Sun et al., 2016). The majority of published human studies have been retrospective and observational in nature and there is some suggestion of small deficits in language and cognition associated with anesthetic exposure at a young age; however, the results need to be interpreted with caution (DiMaggio et al., 2009; Flick et al., 2011; Ing et al., 2012; O'Leary et al., 2016).

Several large human studies have been published recently. A multicenter prospective randomized controlled trial comparing neurodevelopmental outcomes at 2 years of age after general anesthesia or awake regional anesthesia found no evidence that less than 1 h of sevoflurane anesthesia in infancy for inguinal hernia repair increases the risk of adverse neurodevelopmental outcome when compared with awake regional anesthesia (Davidson et al., 2016). The 5 year outcome data for this study is still pending. The PANDA study was a sibling matched cohort study of 105 sibling pairs between the ages of 8 and 15 years (Sun et al., 2016). One sibling had a single general anesthetic exposure at less than 36 months of age for a hernia repair and the other sibling had no exposure. There was no statistically significant difference in global cognitive function, measured by IQ, between the two groups (Sun et al., 2016). Three recent population based studies have found only subtle differences in developmental outcomes in children exposed to anesthetics at a young age (Graham et al., 2016; O'Leary et al., 2016; Glatz et al., 2017). An Ontario group matched children who had surgery under general anesthesia before Early Development Index (EDI) testing was performed around the age of 5 with control children who had no general anesthesia exposure (O'Leary et al., 2016). There was a very small difference between the two groups in incidence of developmental vulnerability (any EDI in the lowest 10%), with 25.6% in the exposed group and 25.0% in the unexposed group (O'Leary et al., 2016). A Manitoba study matched children who had surgery prior to age 4 with control children (Graham et al., 2016). There was a small difference between the two groups with exposed children doing worse in communication/general knowledge and language/cognition domains. The risk was greater in older children at the time of general anesthetic exposure and there was no difference between single and multiple exposures. The third study compared children in Sweden who had undergone one anesthetic at less than 4 years of age with controls (Glatz et al., 2017). There was a very small difference between the groups with an anesthetic exposure before age 4 associated with a mean difference of 0.41% lower school grades and 0.97% lower IQ test scores. There has been some suggestion that children exposed to multiple general anesthetics at a young age may have an increased risk of learning difficulties, but additional studies are needed to provide conclusive evidence (Wilder et al., 2009; Flick et al., 2011; Graham et al., 2016; Glatz et al., 2017).

At this point, it remains difficult to make meaningful conclusions from the current human data. Future studies in both humans and animal models are urgently needed to better understand the actual clinical impact in pediatric patients and to further elucidate the anesthetic effects on neurons and glia with respect to synaptic plasticity, neuronal circuitry, and cytotoxicity. Understanding the specific mechanisms of anesthetic-induced injury will help to delineate ways to mitigate this damage and to develop potential protective strategies.

## The search continues for better and less toxic anesthetic agents

Recent studies are being conducted to identify medications or strategies to reduce any negative effects of exposure to the existing anesthetic agents. Dexmedetomidine, a selective alpha_2_-adrenergic receptor agonist with sedative and analgesic properties, is a newer agent that has recently shown some promise in this regard. Preliminary studies have suggested that dexmedetomidine provides neuroprotection and can reverse the cytotoxic effects of volatile anesthetic agents (Su et al., 2015; Zhou et al., 2015; Alam et al., 2017). Although this agent shows promising effects, additional studies are needed to confirm its neuroprotective effects in the clinical setting.

When tested at the presynaptic level, dexmedetomidine acts as an alpha_2_-adrenergic receptor agonist (Tachibana et al., 2012), and prevents the subsequent release of neurotransmitters like glutamate, noradrenaline, and norepinephrine (Ihalainen and Tanila, 2002; Zhou et al., 2015). The activation of alpha_2_-adrenergic receptors by dexmedetomidine inhibits transmitter release through the suppression of voltage-gated Ca^2+^ channels (Chiu et al., 2011). Dexmedetomidine also results in an overall hyperpolarization of the membrane potential due to activation of K^+^ channels by G-protein coupled receptors (Shirasaka et al., 2007; Ishii et al., 2008). However, dexmedetomidine's neuroprotective role at the level of single synapses remains unknown, as intracellular recordings from synaptically paired neurons exposed to dexmedetomidine cannot be obtained directly in mammalian models.

**Figure 2:**
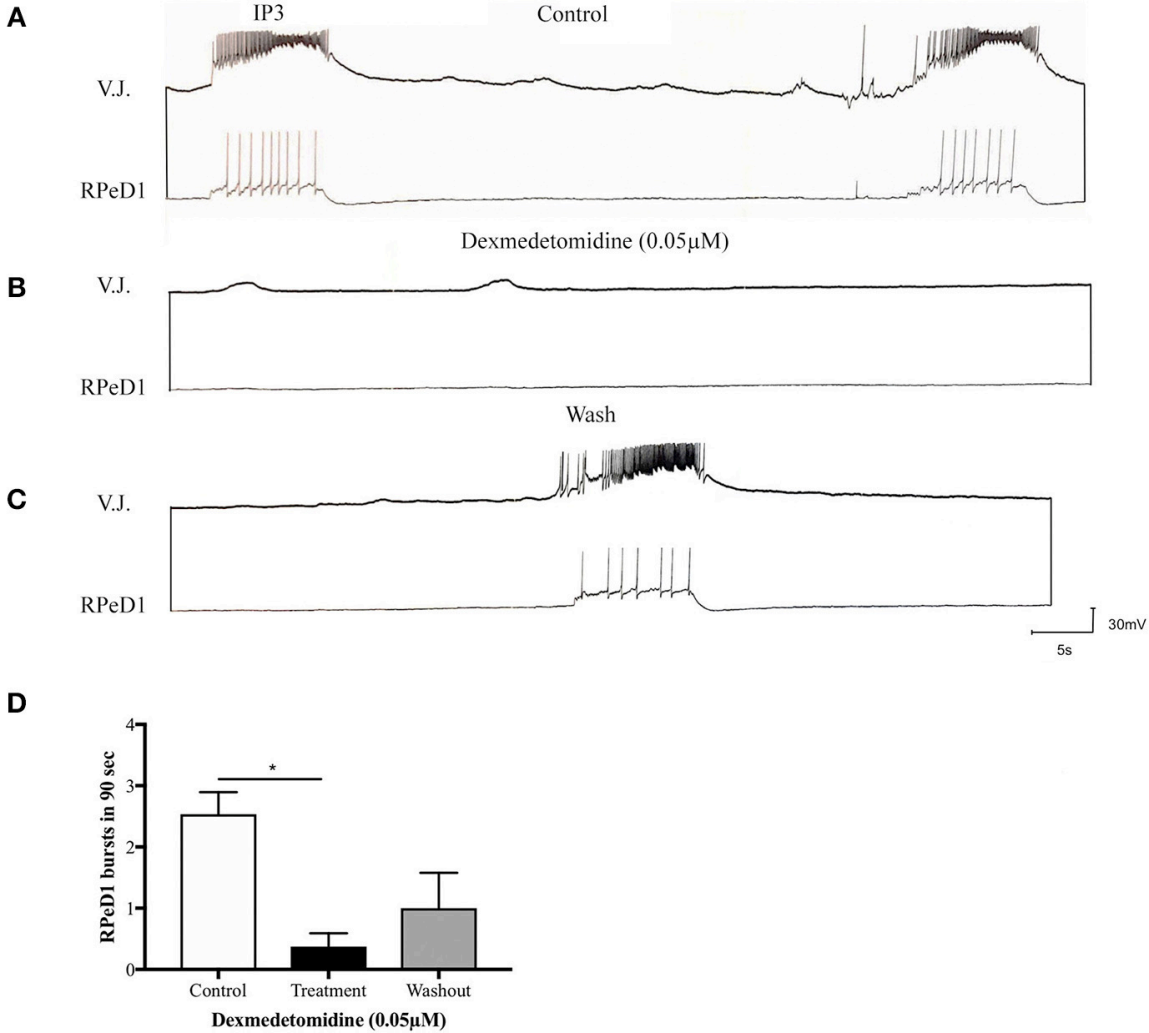
Dexmedetomidine (0.05 μM) blocks spontaneously occurring, respiratory central pattern generator (CPG) activity in isolated *Lymnaea* brain preparation. **(A)** Isolated central ring ganglia of *Lymnaea* exhibits spontaneous, fictive respiratory patterned activity in identified neurons RPeD1 and the VJ cell when recorded intracellularly. These rhythmical discharges are generated by the IP3 interneuron, and have been well-characterized previously. **(B)** When the preparation was bathed in dexmedetomidine (0.05 μM), our preliminary data suggests that the spontaneous rhythmical bursting was blocked. **(C)** The patterned activity began to return upon wash out with normal saline. **(D)** The bar graph presents summary preliminary data suggesting a significant inhibition of respiratory rhythm through cessation of RPeD1 bursting activity during dexmedetomidine treatment. The bursting activity partially restarts upon immediate washout with normal saline. Repeated measure ANOVA was performed with data from three sample preparations. ^*^p < 0.05, error bars ± SEM.

**Figure 3:**
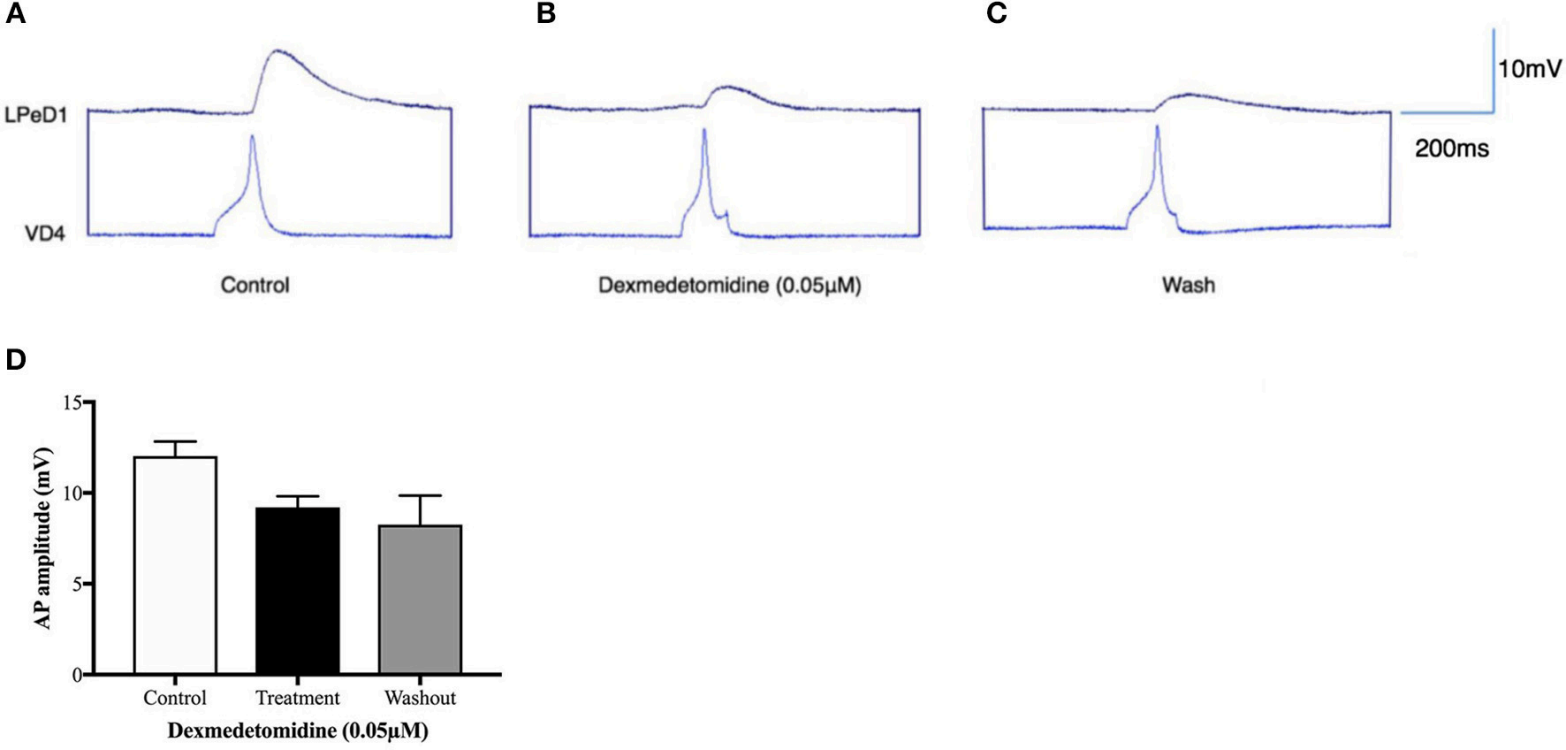
Dexmedetomidine alters synaptic transmission between soma-soma paired *Lymnaea* neurons. Identified neurons were cultured overnight in a soma-soma configuration and simultaneous intracellular recordings were made between presynaptic neuron VD4 and its postsynaptic partner LPeD1 on day two. Induced action potentials in VD4 generated 1:1 excitatory postsynaptic potentials (EPSPs) in LPeD1 (control, **A**). Our preliminary data suggests that the amplitude of the EPSPs in LPeD1 may be reduced in the presence of dexmedetomidine **(B)**. The synaptic transmission did not return to its base line level after 15 min of wash out with normal saline **(C)**. Bar graphs **(D)** present summary preliminary data. Repeated measure ANOVA test on a sample size of three preparations. Error bars ± SEM. AP is an abbreviation of action potential.

Our group thus conducted experiments using *Lymnaea* to examine the effects of dexmedetomidine at the level of single synapses. The following conclusions are speculative and are based on preliminary data. Firstly, our group asked whether dexmedetomidine blocks spontaneous rhythmical bursting of respiratory central pattern generating (CPG) neurons *in vitro* (unpublished observations). Central ring ganglia of *Lymnaea* were isolated *in vitro* and concurrent intracellular recordings were made from the respiratory CPG neurons Right Pedal Dorsal 1 (RPeD1) and the motor neuron Visceral J Cell (VJ) as described previously (Kyriakides et al., 1989; Syed and Winlow, 1989). Our preliminary data indicates that the isolated ganglia exhibited spontaneous respiratory rhythm, which is blocked by dexmedetomidine within minutes of exposure (Figures 2A,B). These effects were reversible and the spontaneous rhythmical pattern returned within minutes of washout with normal saline (Figure 2C). Figure 2D shows quantification of these results. Next, we sought to determine whether dexmedetomidine also blocks synaptic transmission between the cardiorespiratory neurons Left Pedal Dorsal 1 (LPeD1) and Visceral Dorsal 4 (VD4) when paired in a soma-soma configuration. Neurons were paired overnight in a soma-soma configuration (Figure 3) and simultaneous intracellular recordings were made after 18–24 h. Induced action potentials in VD4 (presynaptic) generated 1:1 excitatory potentials in its cholinergic, postsynaptic partner (Figure 3A). This preliminary data allowed us to hypothesize that the cholinergic, excitatory synaptic transmission between the paired *Lymnaea* neurons may be altered within minutes of dexmedetomidine exposure of the preparation (Figures 3B–D).

**Figure 4:**
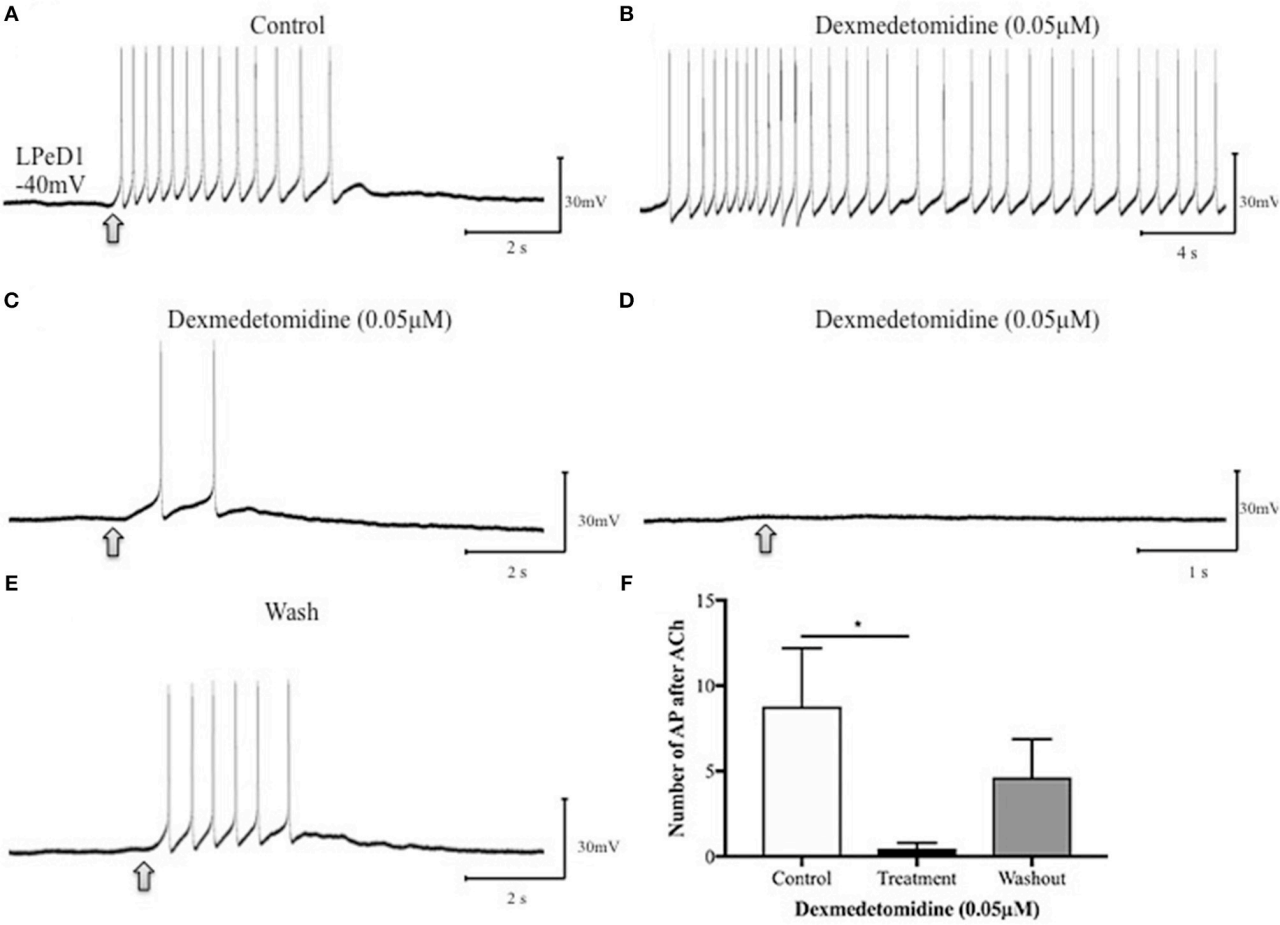
Dexmedetomidine (0.05 μM) blocks extra-synaptic, excitatory cholinergic receptors in the identified *Lymnaea* neuron LPeD1. LPeD1 neurons were isolated *in vitro* and maintained in culture for 18–24h in the presence of brain-conditioned medium (CM). On day two, neurons were impaled with intracellular sharp electrodes and ACh was pressure applied onto the somata, either in the absence or presence of dexmedetomidine. **(A)** LPeD1 neurons exhibited an excitatory response to exogenously applied ACh (1 μM, ~3–6 s, 15-PSI—at arrow) in CM (*n* = 5) held below the firing threshold at −40mV. **(B)** Our preliminary data suggests induction of spontaneous activity in LPeD1 neurons immediately after their exposure to dexmedetomidine (0.05 μM). **(C)** ACh application to LPeD1 after 5 min of dexmedetomidine exposure significantly reduced the excitability to ACh. **(D)** Excitatory cholinergic response in LPeD1 was completely blocked by dexmedetomidine within 10–15min of exposure. **(E)** Upon wash out of the dexmedetomidine with normal saline, the cholinergic response recovered. **(F)** The bar graph presents summary preliminary data based on the number of action potentials occurring after ACh application before returning to baseline before dexmedetomidine exposure, during exposure (after 5 min of dexmedetomidine exposure, as in **C**), and after washout. These preliminary data suggest that dexmedetomidine may block excitatory nAChRs in LPeD1. Repeated measure ANOVA test on a sample size of five preparations. ^*^p < 0.05, error bars ± SEM. AP is an abbreviation of action potential.

**Figure 5:**
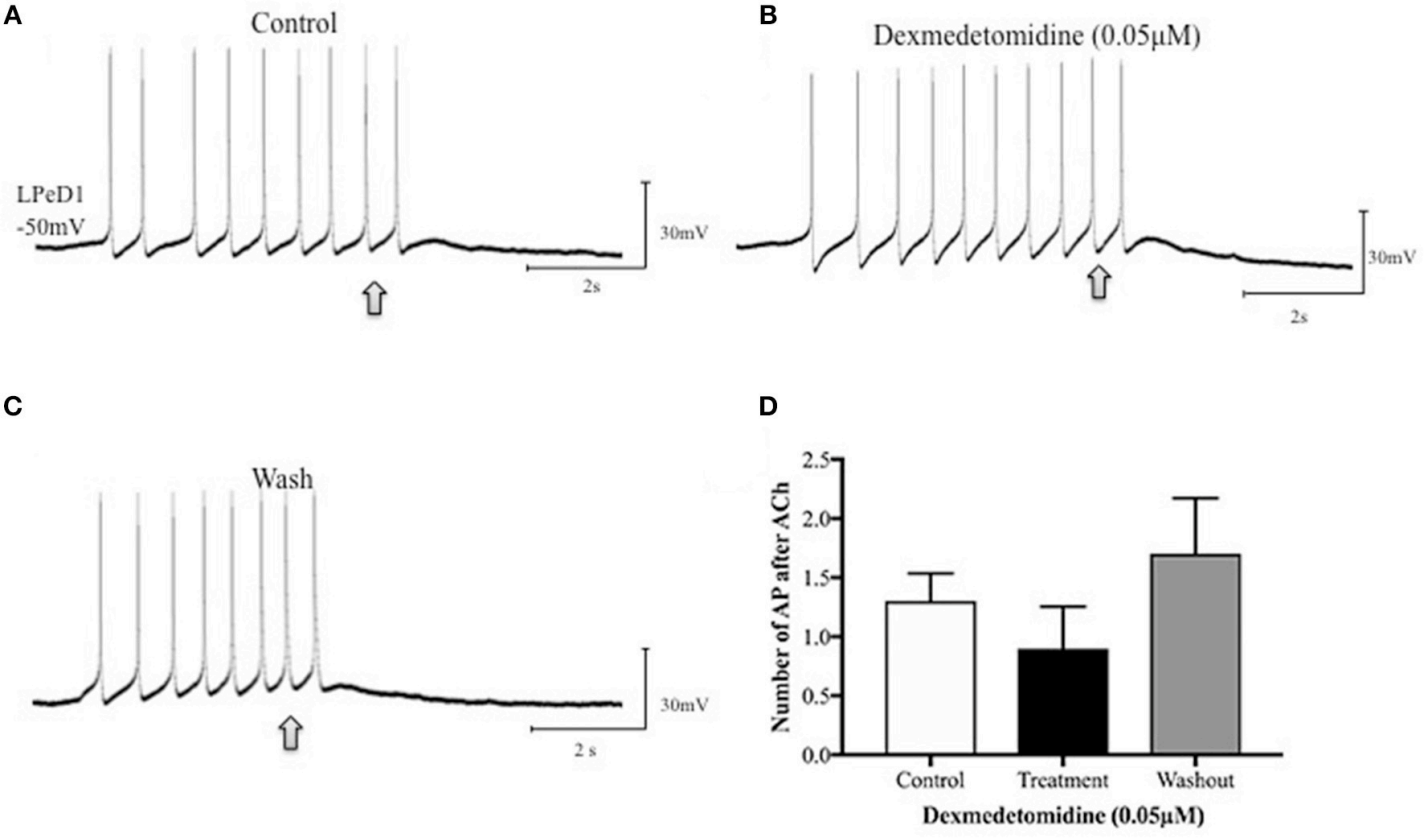
Dexmedetomidine (0.05 μM) does not block inhibitory cholinergic receptors in the identified *Lymnaea* neuron LPeD1. When cultured in defined medium alone (DM—does not contain trophic factors), the LPeD1 neurons exhibit an inhibitory response to exogenously applied ACh. Neurons were isolated in culture and intracellular recordings were made from LPeD1 after 18–24h. All neurons, when held near or below their firing threshold (−40mV), exhibited an inhibitory response to exogenously applied ACh, which prevented continued neuronal spiking. **(A)** Spontaneous firing in LPeD1 was blocked by a single puff of ACh (at arrow). **(B)** Neurons were then exposed to dexmedetomidine for 10–15 min and ACh was applied again (at arrow). Our preliminary data suggests that dexmedetomidine failed to block the inhibitory response of LPeD1 neurons to ACh. **(C)** After several minutes of washout with normal saline, neurons continued to exhibit an inhibitory response to exogenously applied ACh. **(D)** The bar graph shows summary preliminary data of the number of spontaneous action potentials occurring after ACh application until returning to baseline, indicating that dexmedetomidine may not significantly impair inhibitory cholinergic receptors. Repeated measure ANOVA test performed using three preparations. Error bars ± SEM. AP is an abbreviation of action potential.

To further determine whether the dexmedetomidine-induced effects may have involved the postsynaptic cholinergic receptor, single LPeD1 were maintained *in vitro* overnight and acetylcholine (ACh) was applied exogenously via pressure pulses (Luk et al., 2015). When cultured in brain conditioned medium (medium containing trophic factors), ACh excited LPeD1 neurons (Figure 4A). These non-synaptic responses appear to be reversibly blocked by dexmedetomidine (Figures 4B–F). However, when cultured in defined medium (medium with no added trophic factors), the LPeD1 neurons predominantly exhibited an inhibitory response. This response appeared to remain unperturbed by dexmedetomidine (Figure 5), although this finding requires further study.

Taken together, our preliminary data from *Lymnaea* allows us to speculate that dexmedetomidine blocks excitatory, cholinergic synaptic transmission between paired neurons directly and that these effects likely involve excitatory postsynaptic, but not the inhibitory postsynaptic, receptors. Due to the preliminary nature of this data, additional studies are required to validate these findings and to further examine how dexmedetomidine, or other similar agents, could perhaps serve a neuroprotective role, when used alone or in combination with other anesthetic agents. Invertebrate models are uniquely suited to help guide these future, fundamental, experiments.

## Conclusions

From initial studies on the effects of anesthetics in *L. forbesi*, to more recent work with other mollusc species, including the highly versatile *L. stagnalis*, it is clear that molluscs continue to serve as valuable organisms for fundamental basic science anesthetic research. Molluscs offer a solution to the anatomical and experimental limitations posed by mammals as they better enable researchers to isolate various factors (e.g., network complexity, glial influences) in simplified neuronal systems. As such, researchers are able to define the effects of anesthetic compounds on brain function—from single ion channels, to neuronal intrinsic and synaptic properties at a resolution not approachable elsewhere. For instance, the giant squid axon allowed researchers to directly observe possible mechanisms of anesthetic agents that underlie nervous system suppression by inhibiting action potential generation and propagation. In addition, the large, easy to manipulate, and individually identified neurons of the *L. stagnalis* allowed for the discovery of proteins (specifically the nAChR) as a potential site of action for general anesthetic agents rather than solely the cell membrane. In particular, studies in *Lymnaea* have shown that anesthetics induced the inhibition of synaptic transmission in both cholinergic and peptidergic synapses at both excitatory and inhibitory synapses. In terms of anesthetic-induced neurodegeneration and neurotoxicity, results from *Lymnaea* are equivocal. Studies have reported reductions in cholinergic synapse formation, yet only with chronic exposure. In addition, general anesthetics have no effect on post-tetanic potentiation or neurite growth in *Lymnaea* neurons. However, despite this lack of documented effect of anesthetic agents on short-term memory, ketamine has been shown to impair long-term memory in *Lymnaea*. In addition, local anesthetics have been shown to cause neuron apoptosis and arrest neurite growth.

A wealth of work in mammals and in the clinical setting has highlighted that possible negative effects of anesthetics can be neither ignored nor ruled out. As such, research into other compounds that can be used either alone or as adjuvants to general anesthetics that may offer less potential for neurotoxicity is key to moving this field forward. Here, we presented preliminary data on the mechanism of action of one such compound, dexmedetomidine. Additional research is needed to determine if and how this compound might be used to mitigate anesthetic-related neurotoxicity.

## Author contributions

All authors wrote and edited the manuscript. SR, SH, and NS performed experiments.

### Conflict of interest statement

The authors declare that the research was conducted in the absence of any commercial or financial relationships that could be construed as a potential conflict of interest. The reviewer GP and handling Editor declared their shared affiliation.
